# South Africa and its COVID-19 prohibition predilection

**DOI:** 10.3126/nje.v10i3.31543

**Published:** 2020-09-30

**Authors:** Indrajit Banerjee, Jared Robinson, Brijesh Sathian, Edwin R. van Teijlingen

**Affiliations:** 1 Department of Pharmacology, Sir Seewoosagur Ramgoolam Medical College, Belle Rive, Mauritius; 2 Sir Seewoosagur Ramgoolam Medical College, Belle Rive, Mauritius; 3 Geriatric and long term care Department, Rumailah Hospital, Hamad Medical Corporation, Doha, Qatar; 4 Centre for Midwifery, Maternal and Perinatal Health, Bournemouth University, Bournemouth, United Kingdom

## Background

The year 2020 will forever be marked by the COVID-19 pandemic [1]. The virus, which originated in China in 2019, spread across the globe by the Spring of 2020 [2-4]. As of March 11, 2020, the COVID-19 was declared a pandemic by the WHO (World Health Organization) [3]. High, middle and low-income countries alike have been adversely affected by the virus [5]. Globally, as of the 15^th^ of September 2020, there have been 29,155,581 confirmed cases of COVID-19, including 926,544 deaths reported to the WHO [6].

### COVID-19 in South Africa:

South Africa, is a unique country in terms of its geopolitical position as well as its disparities in standards of living. It is classified as a developing country [7]. The healthcare system in South Africa is a mixture of private and government services. The two systems exist in stark contrast to one another [8, 9]. According to the Department of Health in South Africa by September 15, 2020 a total of 3,940,217 tests have been conducted. With 651,521 positive cases identified, 583,126 reported recoveries and a total of 15,641 deaths [10].

There is disparity in the current status of confirmed COVID-19 cases across South Africa’s nine provinces. There have been a total of 651,521 confirmed cases; the provincial breakdown of the cases is as follows: 1) The Gauteng province has the highest number of cases with 215,481 COVID-19 positive patients; 2) KwaZulu-Natal ranks second with 116,674 cases; 3) The Western Cape ranks third with 108537 cases; 4) Eastern Cape has 87,514 cases; 5) The Free State ranks fifth with 42,255 cases. 6) the Northern West records 27,321 cases; 7) Mpumalanga ranks seventh with 25860 cases; 8) Limpopo records 14,263 cases; and 9) The Northern Cape has the lowset number of 13,616 positive COVID-19 cases [10].

As of the 15th September, 2020, South Africa has lost a total of 15,641 lives due to COVID-19 and recorded a total of 583,126 recoveries [10]. Gauteng Province has had the greatest amount of recoveries with 191,668 people recovering from the virus; it has had a total death count of 3,933 which is the second highest provincial loss of lives after the Western Cape Province with 4,079 deaths and 101,044 recoveries. The highest number of active COVID-19 positive cases (19,880 cases) have been reported from Gauteng Province. KwaZulu-Natal has the second highest amount of recoveries with 107,149 patients being cured and deaths totaling 2,425. The Eastern Cape has had recoveries totaling to 83,629 and has lost 3,055 lives. The Northern Cape has had the both the least recoveries 10,412 and the fewest deaths (n=176).

### Governance and response to COVID-19:

The South African government have introduced a range of parameters and laws in order to curb the spread of the virus whilst simultaneously endorsing programs to spearhead the preparedness of the healthcare system for the various waves of COVID-19 cases that have been forecast. The government has used lockdown legislation and restriction of movement bills to curb the transmission of the disease. A unique method employed by the government was the prohibition of alcohol and tobacco products to protect people from infection [11-12]. The construction of multiple field hospitals, mobile testing units, and personal protective equipment bills are in the government’s arsenal to fight the virus, with further preparedness indictated by the digging of 1.2 million graves [13].

### Lockdown legislation:

In conjunction with the new laws and regulations, the Government of South African has exercised lockdown and restriction of movement policies. These policies being implemented on the basis of various levels of severity ranging from the highest alert level 5 through to alert level 1. Level 5 to 2 have each a unique set of rules and regulations with the primary aim of curbing the transmission of the virus.

### Alert level 5:

This is the most stringent level with extreme limitations to the movement of people. Only essential services are granted operational capability. In the transport sector taxis, buses and private motor vehicles are only allowed to be operational in designated hours and strict occupancy rules are to be followed. Under level 5 no interprovincial travel is permitted. The sale of alcohol and tobacco products is prohibited. The use of face masks is mandatory. Non-essential retail businesses must stay closed [13, 14].

### Alert Level 4:

All essential services as well as all financial and professional services are allowed to be operational. Stores may sell non-essential goods that are within their existing inventory. Waste recycling is permitted, all types and forms of agricultural activity as well as global services for export markets are operational. The information technology, the postal and telecommunication services are permitted to be operational. The mining sector is permitted to have opencast mines operating at 100% capacity, other forms of mining are restricted to 50%. In addition to this transport services may operate at any time of the day, under the guidance of strict hygiene standards. No interprovincial movement is permitted unless under vetted circumstances. Every citizen must stay at their place of residence from 8pm to 5am. Walking and jogging are permitted, but not in groups [13, 14].

### Alert Level 3:

Students may return to school or tertiary education facilities. The visiting of family is prohibited. Restaurants are allowed to operate with eat-in meals on a reduced capacity basis following strict health regulations. Hairdressers and personal services of similar nature are allowed to be operational, conferences are permitted for work purposes, hotels and commercial accommodation centres may be operational. Cinemas, theatre’s and casinos are operational with limited capacity. Non-contact sports such as golf and tennis are permitted under the condition that the necessary health regulations are adhered to [13, 14].

### Alert Level 2:

Restaurants may remain open, with a curfew between 10pm and 4am. The prohibition of alcohol is now disband and alcohol may be served in licensed establishments. The restrictions on guests and social gatherings remain in place. Interprovincial travel is permitted. Visits to family and friends in small groups is permitted. All retail establishments may open and all government sectors are to be operational. All mining activities may operate at 100% capacity. Construction and all other forms of manufacturing is permitted. Domestic work and cleaning services are permitted. South Africa is currently under alert level 2 regulations [13, 14].

### Alert level 1:

Restrictions have not yet been stipulated.

### Outcome of the stringent prohibition policies:

Although the prohibition of alcohol and tobacco has sent shock waves through this liberal African country. It is a stance many other governments would not dare to exercise due to public opinion, pressure from sellers/producers of alcohol and tobacco and a host of intrinsically attached difficulties [15]. The prohibition of alcohol in South Africa has been an empirically effective tool in reducing the burden of casualty departments in government healthcare facilities across the country. A study from the Province of Kwa-Zulu Natal in April 2020 showed a 47% reduction in severe trauma cases [16]. This massive reduction decreases the burden on the already inundated Government health facilities and frees up resources and staff to handle COVID-19 cases [17]. The prohibition of alcohol alone is a considerable factor in preparing the system for the influx of the COVID-19 cases.

### The toll of alcohol on South African citizens:

Deaths induced by alcohol as well as alcohol-associated deaths account for the loss of 62,300 lives annually in South Africa [12]. These alcohol-related deaths spans from loss of lives due to intoxication, road traffic accidents, assaults, rape and gunshot injuries. A study by Reuter and colleagues stresses the positive effects of the prohibition of alcohol on the health care system [12]. Policies of such a stringent nature have been applauded by numerous papers such as by Matzopoulus and team, who believe that the alcohol ban bodes a positive potential for improved and better policy making in future [18].

### Practical implication of prohibition policies:

By virtue of simple policy changes and amendments, a stark reduction can be seen on the burden of healthcare systems. The implementation of the prohibition principle may save the government millions, if not billions, as well as raise “COVID-19 preparedness” as existing resources will be freed up to combat the virus; whilst simultaneously saving people’s lives.

South Africa’s unique policy intervention may therefore become a global pioneer in future policy development and crisis management strategies for other governments who need to reduce the burden on their healthcare systems for an impending influx of patients. Ultimately it is a simple principle, which employs strong policy making and legislation, whilst dually safeguarding the lives of citizens. It allows for resources to be managed in a more precise and useful manner which ultimately benefiting many in society.

### Timeline of COVID-19 in South Africa

The first confirmed COVID-19 positive case was reported on the 5^th^ of March 2020, a 38-year-old male patient who had been to Italy [19]. Daily reported cases grew steadily until May 2020, and from June 2020 onwards cases rose sharply showing an exponential trajectory [19]. South Africa surpassed 100,000 cases in June 2020 and then 200,000 in July. As of the 2nd of July 2020, the number of cases increased at a higher rate and produce the steep trajectory. The daily number of new cases has declined rapidly from approximately 13,500 daily cases in mid of July to 2,000 cases per day in the first week of September.

### Rehabilitation concern for COVID-19

Rehabilitation COVID-19 cases should consider (a) optimizing outcomes of people with severe COVID 19 and (b) the services for people with more essential or basic needs.

Gaps and challeges in Southern Africa are real time onsite training challenges, cascade trainings of all health workers, health care worker getting infected, a general shortage of health care facility and staff, and limited surge capacity.

Across the globe older people and those with co-morbidities and/or disabilities are more likely to suffer impairments due to COVID-19. Severe cases of COVID-19 often need rehabilitation after ventilator support, prolonged immobilization and bed rest. Patients with COVID-19 who do not receive invasive mechanical ventilation, either because these resources are not available or where illness severity does not warrant this, may also experience some degree of physical or respiratory impairment, as well as psychosocial challenges, as a result of the illness and hospitalisation [3]. Respiratory disorders and lack of exercise in the elderly can lead to diseases such as apraxia syndrome and pulmonary infections.

Little is known about long-term effects/impairments of COVID-19 therefore patient follow-up is vital. Community engagement is a key element due to stigmatization of people who have been hospitalized with COVID-19 [20]. Countries will require increased surge capacity - this may not match the capacities of their rehabilitation staff– the unmet rehabilitation needs/lack of resources needs to be flagged to health ministries. IPC infection prevention and control) measures, and access to appropriate PPE (personal protective equipment) are essential. Modifications relating to how rehabilitation is delivered will be required for infection control – Telehealth may need to be developed to support staff in their work and their training needs. Mental wellbeing support for staff should be strengthend. A multi-disciplinary approach is key, and so is the sharing of new learning internationally, whilst keeping up-to date with WHO advice and guidance. Rehabilitation interventions must match skill sets of rehabilitation professionals. To make the best use of telehealth, areas that are resource poor need to be addressed at the service/government level.

## Conclusion

South Africa’s unique, multifaceted and strategic method to combatting the coronavirus has proven to be effective in using existing resources and redirecting human and other resources. The prohibition of alcohol is a unique method employed by the government, the full extent to which this policy reform has benefitted the country is still to be determined as South Africa’s coronavirus cases are still increasing. Thusfar, reports suggest that the prohibition of alcohol has been greatly beneficial. This policy reform may become popular as part of crisis management protocols in other countries if its long-term benefits can be established.

## References

[1] Guo YR Cao QD Hong ZS Tan YY Chen SD Jin HJ Tan KS Wang DY Yan Y The origin, transmission and clinical therapies on coronavirus disease 2019 (COVID-19) outbreak - an update on the status. Mil Med Res. 2020 3 13;7(1):11. https://doi:10.1186/s40779-020-00240-0 PMid: ; PMCid: . 32169119 10.1186/s40779-020-00240-0 PMC7068984

[2] Banerjee I Mohabeer P Shukla A Kashyap A Robinson J COVID-19: Recent advances in epidemiology, virology, etiopathogenesis, clinical trials and vaccine development. J Biomed Sci, 2020; 7(1), 18-27. 10.3126/jbs.v7i1.2984

[3] Chen N Zhou M Dong X Qu J Gong F Han Y Qiu Y Wang J Liu Y Wei Y Xia J Yu T Zhang X Zhang L Epidemiological and clinical characteristics of 99 cases of 2019 novel coronavirus pneumonia in Wuhan, China: a descriptive study. Lancet. 2020 2 15;395(10223):507-513. https://doi:10.1016/S0140-6736(20)30211-7 PMid: ; PMCid: . 32007143 10.1016/S0140-6736(20)30211-7 PMC7135076

[4] Tu H Tu S Gao S Shao A Sheng J Current epidemiological and clinical features of COVID-19; a global perspective from China. J Infect. 2020 7;81(1):1-9. http://doi:10.1016/j.jinf.2020.04.011 PMid: ; PMCid: . 32315723 10.1016/j.jinf.2020.04.011 PMC7166041

[5] Banerjee I Robinson J Kashyap A Mohabeer P Shukla A Leclézio A The changing pattern of COVID-19 in Nepal: A Global concern- A Narrative Review. Nepal J Epidemiol. 2020 6 30;10(2):845-855. https://doi:10.3126/nje.v10i2.29769 PMid: ; PMCid: . 32874698 10.3126/nje.v10i2.29769 PMC7423402

[6] WHO Coronavirus Disease (COVID-19) Dashboard. [online 2020] [cited 2020 Sept15] Available from: URL: https://covid19.who.int

[7] Peltzer K Phaswana-Mafuya N Pengpid S Rural-urban health disparities among older adults in South Africa. Afr J Prim Health Care Fam Med. 2019 6 19;11(1):e1-e6. https://doi:10.4102/phcfm.v11i1.1890 PMid: ; PMCid: . 31296012 10.4102/phcfm.v11i1.1890 PMC6620551

[8] Dell AJ Kahn D. Klopper J Surgical resources in South Africa: an analysis of the inequalities between the public and private sector. S Afr J Surg. 2018 6;56(2):16-20. 10.17159/2078-5151/2018/v56n2a2397 PMid: . 30010259

[9] van Rensburg HC South Africa’s protracted struggle for equal distribution and equitable access - still not there. Hum Resour Health. 2014 5 8;12:26. https://doi:10.1186/1478-4491-12-26 PMid: ; PMCid: . 24885691 10.1186/1478-4491-12-26 PMC4029937

[10] COVID-19 Corona Virus South African Resource Portal. Press Releases and Notices. [online 2020] [cited 2020 Sept 15] Available from: https://sacoronavirus.co.za/category/press-releases-and-notices/

[11] Wadvalla BA. How Africa has tackled covid-19. BMJ. 2020 7 16;370:m2830. https://doi:0.1136/bmj.m2830 PMid: . 32675053 10.1136/bmj.m2830

[12] Reuter H Jenkins LS De Jong M Reid S Vonk M Prohibiting alcohol sales during the coronavirus disease 2019 pandemic has positive effects on health services in South Africa. Afr J Prim Health Care Fam Med. 2020 1 1;12(1):1-4. 10.4102/phcfm.v12i1.2528 PMid: PMCid: 32787395 PMC7433289

[13] Regulations and Guidelines - Coronavirus COVID-19. South African Government. [online 2020] [cited 2020 Sept 15] Available from: URL: https://www.gov.za/coronavirus/guidelines

[14] Arndt C Davies R Gabriel S Harris L Makrelov K Robinson S Levy S Simbanegavi W van Seventer D Anderson L Covid-19 lockdowns, income distribution, and food security: An analysis for South Africa. Glob Food Sec. 2020 9;26:100410. https://doi:10.1016/j.gfs.2020.100410 PMid: ; PMCid: . 32834955 10.1016/j.gfs.2020.100410 PMC7366977

[15] Dyer O. Covid-19: Africa records over 10 000 cases as lockdowns take hold. BMJ. 2020 4 8;369:m1439. https://doi:10.1136/bmj.m1439 PMid: . 32269023 10.1136/bmj.m1439

[16] Morris D Rogers M Kissmer N Du Preez A Dufourq N Impact of lockdown measures implemented during the Covid-19 pandemic on the burden of trauma presentations to a regional emergency department in Kwa-Zulu Natal, South Africa. Afr J Emerg Med. 2020 6 16. https://doi:10.1016/j.afjem.2020.06.005 PMid: PMCid: 32837876 10.1016/j.afjem.2020.06.005 PMC7296321

[17] Maphumulo WT Bhengu BR Challenges of quality improvement in the healthcare of South Africa post-apartheid: A critical review. Curationis. 2019;42(1):e1-e9. https://doi:10.4102/curationis.v42i1.1901 PMid: ; PMCid: . 31170800 10.4102/curationis.v42i1.1901 PMC6556866

[18] Matzopoulos R Walls H Cook S London L South Africa’s COVID-19 Alcohol Sales Ban: The Potential for Better Policy-Making. Int J Health Policy Manag. 2020 6 10. https://doi:0.34172/ijhpm.2020.93 PMid: . 32610805 10.34172/ijhpm.2020.93 PMC7719204

[19] First case of covid-19 coronavirus reported in SA. National Institute for Communicable Diseases. [online 2020] [cited 2020 Sept 15] Available from: https://www.nicd.ac.za/first-case-of-covid-19-coronavirus-reported-in-sa/

[20] Sathian B Asim M Mekkodathil A van Teijlingen E Subramanya SH Simkhada S Marahatta SB Shrestha UM Impact of COVID-19 on community health: A systematic review of a population of 82 million, JAIM 2020;9(1): 4-11. 10.3126/jaim.v9i1.29159

